# Editorial: The potential of a multifactorial perspective on dementia

**DOI:** 10.3389/fpsyt.2025.1612550

**Published:** 2025-04-29

**Authors:** Isabelle van der Velpen, Rabih Chattat, Myrra Vernooij-Dassen

**Affiliations:** ^1^ Department of Radiology and Nuclear Medicine, Erasmus University Medical Center, Rotterdam, Netherlands; ^2^ Department of Psychology, University of Bologna, Bologna, Italy; ^3^ Department of IQ Healthcare, Radboud University Medical Center, Nijmegen, Netherlands

**Keywords:** social health, dementia, prevention, lived experience, cognitive decline

Dementia research is undergoing a paradigm shift from a biological to a multifactorial perspective. The recognition of dementia as a multifactorial disorder has encouraged the exploration of new avenues to understand mechanisms related to onset, progression, and treatment options. The identification of potentially modifiable risk factors for dementia by Livingston et al. (1) and their estimate of a 45% risk reduction through prevention efforts have been a major impetus to find new ways to prevent dementia. This perspective also challenges the paradigm of dementia as a non-avoidable disaster and introduces the new paradigm of a proactive approach to preventing cognitive decline and dementia, and modulating dementia progression. In recent years, the role of social factors in the onset and the progression of dementia has become more important and several studies have outlined the impact of social factors on prevention and care (2–4). In line with this, one of the modifiable dementia risk factors identified by Livingston et al. is social isolation (1). This single marker has been expanded through the umbrella concept of social health, using a framework that has been valuable in discussing and interpreting the wide range of research on social domains in the context of dementia risk, prevention and management. ‘Social health’ is a relational concept in which well-being is defined both as the impact that an individual has on others (social environment), and as the impact of the social environment (others) on the individual (5). Social health has been around since its introduction by the World Health Organization in 1946 as one of the three domains of health, along with physical and mental health. However, the potential of social health remains underused in dementia research and clinical practice. Many studies, including two large multidisciplinary projects, have revealed associations between social health and cognition and dementia risk (6–11). These studies have also delivered potentially modifiable markers of social health risk and protection. After all, social and mental health may play a role in the prevention and clinical management of cognitive decline and dementia. In this series we further explore the social health perspective as part of a multifactorial approach to prevent dementia and to clinically manage dementia-related disability.

This Research Topic aims to contribute to a richer understanding of dementia as a multifactorial disorder. In their perspective paper, Bruinsma et al. described challenges and outlined future (research) endeavors to establish a better operationalization of social activities in multidomain interventions to prevent dementia. They recommended conducting mixed methods research, focusing on the promotion of engagement in social activities outside the intervention setting, and exploring the needs and preferences of older adults for digitally supported interventions through co-design. On the clinical side of the dementia spectrum, qualitative and quantitative studies along with systematic and scoping reviews explored lived experiences (Thijssen et al., Yaron et al.), identified profiles of relationship quality and needs (Marques et al., Vincente-Alba et al.), examined novel markers of social health (Kristanti et al.), and identified current instruments to measure social health markers (Altona et al.), in addition to trends in psychotherapies and psychosocial interventions (Vincente et al.), all applied to persons living with dementia. Two qualitative studies highlighted the perspectives of persons living with dementia and other stakeholders. Thijssen et al. used a co-research design in a qualitative study to gain insight into the perspectives of stakeholders on the involvement of persons living with dementia and their carers in the development and sustainment of dementia-friendly initiatives. Yaron et al. explored how persons living with dementia and their carers understand living well with dementia in an everyday context, highlighting the insider perspective in another qualitative study. Two studies targeted social health markers and instruments, building upon lived experiences. In an international qualitative study, Kristanti et al. pinpointed novel social health markers in the context of dementia, relevant to persons living with dementia and their social environment. The voices at the center of these various studies spoke clearly about the value of interpersonal relationships in different contexts and gave recommendations for researchers and care providers in the dementia space. In a thorough systematic review, Altona et al. identified instruments used to measure social health markers within the context of cognitive functioning, cognitive decline, and dementia, emphasizing the necessity of clear definitions and multidimensional tools for a comprehensive understanding. This was further underscored in the work of Vincente-Alba et al., who examined the relationship between needs and functional capacity and dependency in persons living with cognitive impairment or dementia, recommending the implementation of needs assessments as part of the comprehensive biopsychosocial assessment and person-centered care for persons living with dementia. This focus on needs is further reflected in the work of Marques et al., who aimed to identify and characterize distinct profiles based on relationship quality within a large cohort of persons living with dementia and their informal carers and to explore the factors influencing each profile. Factors contributing to relationship quality profiles are amenable to intervention. The identified profiles may identify individuals at risk for poorer outcomes, and who may therefore benefit most from timely psychosocial interventions. Current trends in psychosocial interventions and psychotherapies were identified in a scoping review by (Vincente et al.). Eight distinct categories of interventions related to various strategies were found. The diversity of options in psychotherapies and psychosocial interventions for persons living with dementia highlights that there is something to choose from for each person’s unique preferences and experiences.

In both dementia prevention and the clinical management of dementia progression, clear definitions, appropriate markers and comprehensive measurement tools are an essential starting point for the incorporation of social health into research, prevention and clinical practice. Understanding and incorporating the perspectives of persons living with dementia and other stakeholders is instrumental in finding effective strategies for definitions, measurement and interventions. The quality of social relationships and their complex dynamics in a context of constant change may contribute to the heterogeneity of dementia progression. In order to apply a proactive approach, the expertise and support of persons living with dementia are crucial.
